# The application of single-port laparoscopic percutaneous internal ring suture for the management of indirect inguinal hernia in female adults

**DOI:** 10.1038/s41598-020-73171-4

**Published:** 2020-10-01

**Authors:** Shih-Hsien Wang, Ju-Bei Yen, Sheng-Lung Hsu

**Affiliations:** 1 Department of Pediatric Surgery, Chang-Gung Memorial Hospital, Chiayi, Taiwan; 2 School of Medicine, Chang-Gung University, Taoyuan, Taiwan; 3 Department of Pediatrics, Chang-Gung Memorial Hospital, Chiayi, 61363 Taiwan; 4 Graduate Institute of Clinical Medical Sciences, Chang-Gung University, Taoyuan, 33302 Taiwan; 5 Department of Diagnostic Radiology, Chang-Gung Memorial Hospital, Chiayi, 61363 Taiwan

**Keywords:** Diseases, Gastroenterology

## Abstract

As most of the female inguinal hernias are of indirect type, we conducted this retrospective study to evaluate whether the single port laparoscopic percutaneous internal ring suture is feasible for the management of indirect inguinal hernia in female adults. From April 2016 to August 2019, there were 31 female adults who were diagnosed with inguinal hernias and received laparoscopic inspection at our surgical department. One patient who was finally diagnosed as an encysted hydrocele was excluded from the statistic study. All the 30 cases were of indirect type with a total of 35 single port laparoscopic percutaneous internal ring sutures performed. The median age was 38 years (range 20–88 years). The number and percentage of patients with right, left and bilateral hernias were 17 (56%), 11 (37%) and 2 (7%) respectively. Three contralateral patent processi vaginalium and 1 occult femoral hernia were found during operation. The percentages of the respective classifications according to the European Hernia Society system for the 35 PIRSs were L1: 40%, L2: 49%, and L3: 11%. The average operation time was 18 min for unilateral and 30 min for bilateral hernias. There were 1 recurrence and 1 chronic postoperative inguinal pain. Both had their symptoms and signs resolved after reoperation. The mean follow-up period was 13.6 months. We concluded that the single-port laparoscopic percutaneous internal ring suture is feasible for the management of indirect inguinal hernia in female adults.

## Introduction

Inguinal hernia (IH) is an uncommon disease for women. Statistically, 27% of males develop IH in their lifetime, while only 3% of females experience IH^1^. However, females are four times riskier than males in suffering from femoral hernias^2^.

Although female IHs are different from the male ones in the incidence and pathophysiology, there has been no consensus about the management of female IHs. According to a nationwide database from the Denmark, as high as 5.2% of the women who received herniorrhaphy needed reoperation^3^. Literatures have revealed that the high postoperative recurrence rate of female IH may link to the misdiagnosis of a primary femoral hernia^4–9^. Furthermore, those surgical techniques widely used on men seemed to be risky for the hernia repair in women^3,9^. The European Hernia Society (EHS) guidelines thus recommend women with groin hernias undergo laparoscopic repair with preperitoneal mesh placement because it covers both the inguinal and femoral hernias^10^.

Laparoscopic-assisted percutaneous internal ring suture (PIRS) had been developed for over two decades in the repair of indirect IH (IIH) for children. The principle of PIRS was to close the internal ring opening (IRO) at the preperitoneal level with a nonabsorbable suture. Literatures have revealed this is an effective technique and is comparable with the traditional pediatric herniorrhaphy in terms of the operation time, complications, and recurrent rate^11–13^. Previous reports have shown that most of the inguinal hernias in females are indirect type^1,14–16^. To apply this technique for the repair of IHs in female adults, we further developed four key steps during the operation to secure the procedure. Here we present our initial experience of PIRS for the management of female IHs.

## Methods

**Figure 1:**
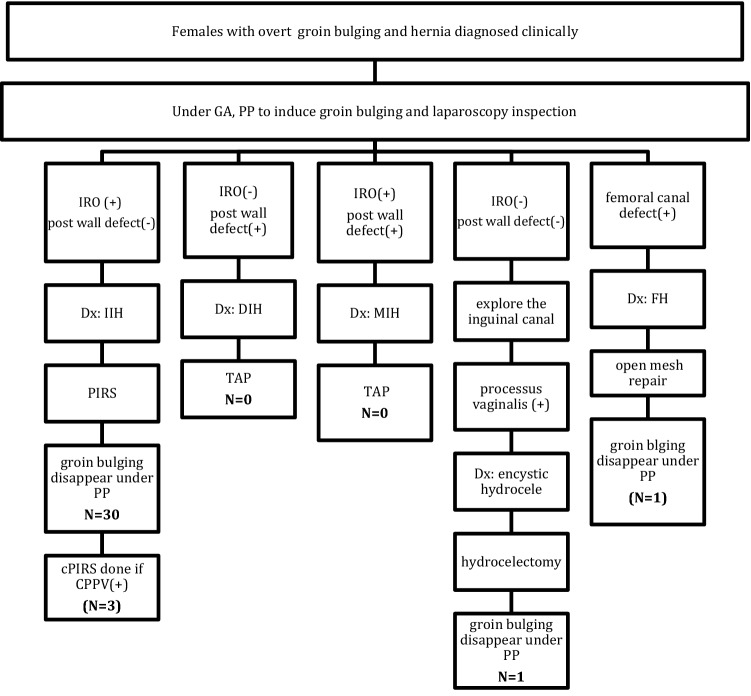
Customizing the treatment of a female inguinal hernia by using laparoscopy. Although various types of conditions have been considered in this flow chart, neither DIH nor MIH was encountered in our series. Parentheses for the numbers of CPPV and FH mean that these were associated findings and were included in the numbers of IIH. *GA* general anesthesia, *PP* pneumoperitoneum, *IRO* internal ring opening, *CPPV* contralateral patent processus vaginalis, *IIH* indirect inguinal hernia, *DIH* direct inguinal hernia, *MIH* mixed inguinal hernia, *TAP* transabdominal preperitoneal mesh repair, *FH* femoral hernia, *cPIRS* contralateral percutaneous internal ring suture.

With the approval of Chang Gung Medical Foundation Institutional Review Board, this retrospective study was conducted through reviewing the medical records of females aged over 20 years who were diagnosed to have IHs at our surgical department. All non-hernial groin bulges, such as those caused by lymphadenopathy, abscess, Bartholin’s cyst or round ligament varices were excluded. For all cases of IH, the diagnosis had been made by patient’s history and the clinical finding of an overt reducible bulge. However, the accurate identification of either a direct (DIH), indirect (IIH), or mixed IH (MIH) could not be obtained before operation. We tried to customize the treatment of IH according to its type. The hernia type was determined under laparoscopy and classified according to European Hernia Society (EHS) system^10^. A standard transabdominal preperitoneal mesh repair (TAP) would be performed if DIH or MIH encountered, while a laparoscopic-assisted PIRS would be proceeded if IIH was confirmed (Fig. 1). The postoperative pain was managed with the oral nonsteroidal anti-inflammatory agent (diclofenac) 25 mg, three times a day for 3 days. All the operations were performed by one surgeon. Written informed consents for the surgical procedure were obtained before surgery. All the authors declared that this study was carried out in accordance with the principles of the Declaration of Helsinki. The characteristics of each patient including age, body weight, body mass index (BMI), onset period of groin bulging, laterality, EHS classification, operation time, and contralateral patent processus vaginalis (CPPV) were documented. The postoperative wound pain, resumption of daily activities, cosmetic satisfaction, and recurrence were followed at our outpatient clinic.

### Surgical procedures

**Figure 2:**
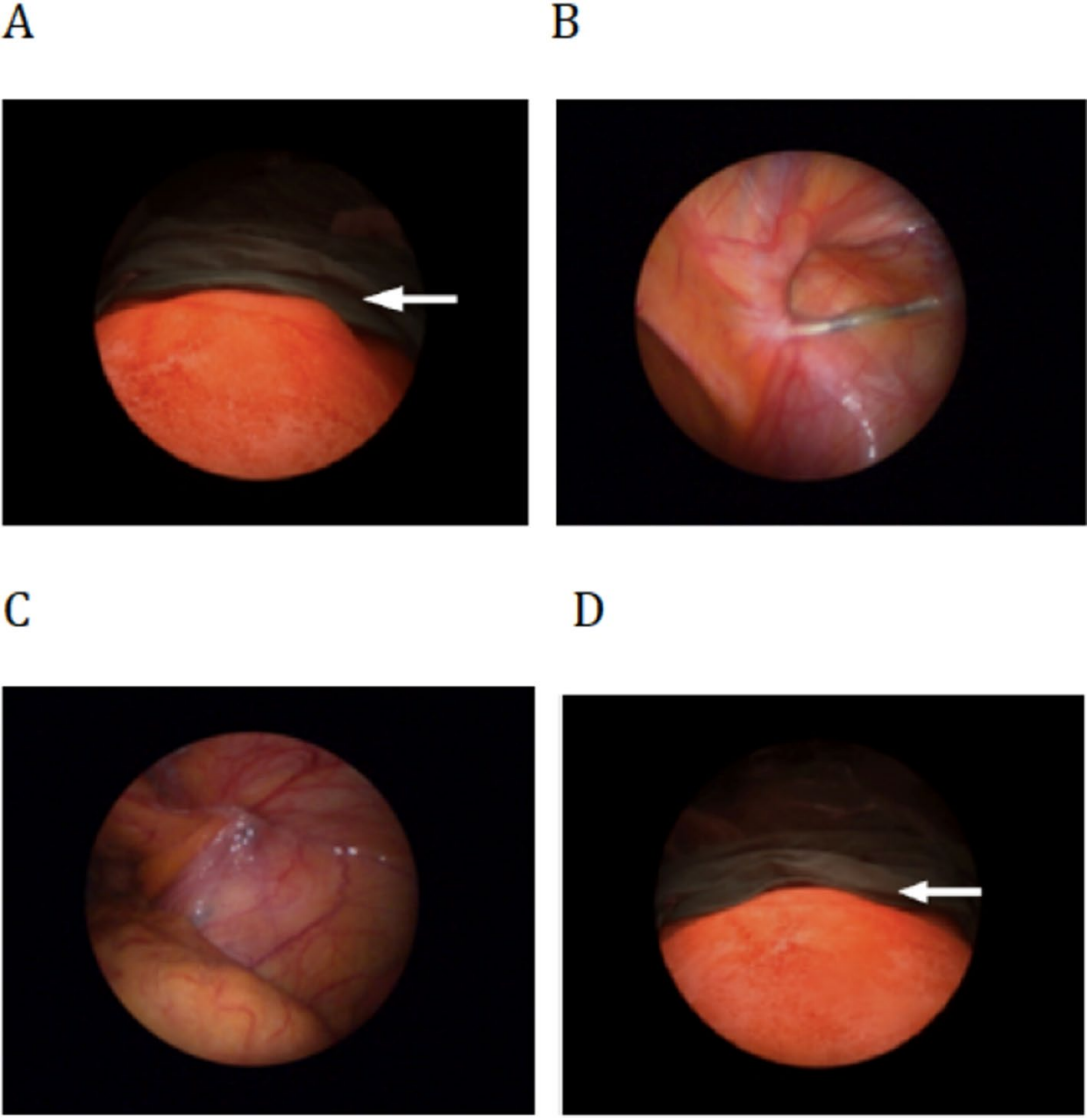
The four key steps in single-port laparoscopic treatment of female inguinal hernias. (**A**) The groin bulge delineating the hernia (white arrow) was induced under a high intra-abdominal pressure of 15 mmHg. (**B**) The scopy showed a right internal ring opening of 2.0 cm in diameter (indicated by the scale over the needle), which confirmed the diagnosis of an European Hernia Society (EHS) L2 hernia. The percutaneous internal ring suture (PIRS) was performed under the single port laparoscopy. (**C**) The internal ring was closed under a low intra-abdominal pressure by tying the non-absorbable thread extracorporeally. (**D**) Finally, the high intra-abdominal pressure of 15 mmHg resumed to confirm the disappearance of groin bulge (white arrow).

After general endotracheal anesthesia, the patient was put in the supine position. By using the single-port laparoscopy, we have developed four key steps (Fig. 2) during the procedure to ensure a reliable treatment of IH.

*Step 1* Pneumoperitoneum and induction of groin bulging: the pneumoperitoneum was created by insertion of a 3 mm trocar into the abdominal cavity directly through the umbilicus and insufflation with CO_2_. At the intra-abdominal pressure of 15 mmHg and flow rate of 6–10 L/m, the groin bulging mimicking the clinical picture of IH was induced (Fig. 2A).

*Step 2* Determination of hernia type: a 3 mm scope was used for the inspection of intra-abdominal wall. The hernia type and its relationship to the induced groin bulging were then determined.

**Figure 3:**
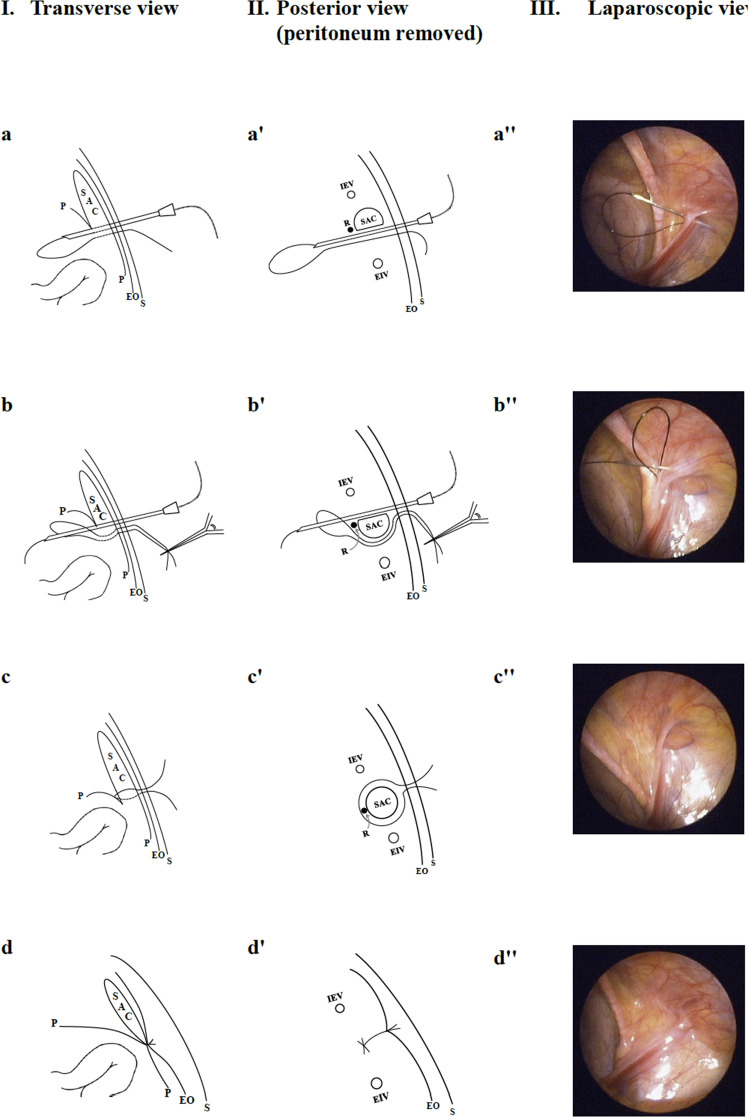
The procedure of percutaneous internal ring suture for female indirect inguinal hernia. (**I**) The transverse section of abdominal wall at the level of inguinal region. (**II**) The posterior abdominal wall viewed from inside the abdomen after the peritoneum was removed. (**III**) The laparoscopic view. a, a′, a″: the needle with a 2–0 Nylon was thrust into the abdominal cavity after passing the lower portion of internal ring opening (IRO). b, b′, b″: a second 2–0 Nylon was introduced to surround the upper portion of IRO and came out from the loop of the first thread. c, c′, c″: the IRO of hernia sac, including the round ligament, was encircled by the 2nd thread at the pre-peritoneal level. d, d′, d″: the IRO was closed by tying the 2nd thread extracorporeally with its knot on the external oblique aponeurosis. *SAC* hernia sac, *P* peritoneum, *EO* external oblique aponeurosis, *S* skin, *IEV* inferior epigastric vessels, *EIV* external iliac vessels, *R* round ligament.

*Step 3* Treatment of IIH by the PIRS technique: if an EHS lateral/indirect type hernia was diagnosed, the PIRS was then performed as is depicted in Fig. 3. A 2–0 Nylon thread carried by an epidural needle (Perifix epidural needle, “B. Braun Melsungen AG”) was pierced into the abdominal wall just lateral to the IRO until the preperitoneal space was encountered. The needle then advanced carefully around the lower hemi-circumferential of the IRO and came out into the abdominal cavity medial to the IRO (Fig. 3a,a′,a″). At the same time, the diameter of IRO was calibrated according to the scale marking over the needle (Fig. 2B). After removing the needle, the 2–0 Nylon was left in situ with its distal loop in the abdominal cavity. Again, with the aid of an epidural needle, a second 2–0 Nylon thread was introduced via the same punctured site, directed to surround the upper hemi-circumferential of IRO at the preperitoneal level, and came out from the loop of the first thread (Fig. 3b,b′,b″). After removing the needle, the 1st looping thread was pulled out of the skin, carrying the end of 2nd thread out of skin simultaneously, resulting in encirclement of the IRO by the 2nd thread at the preperitoneal level (Fig. 3c,c′,c″). The IRO was then closed by tying the 2nd thread extracorporeally (Fig. 3d,d′,d″). Before closing the IRO, the intra-abdominal pressure was decreased to 6 mmHg with a flow rate of 3 L/m to secure the tying (Fig. 2C).

*Step 4* Confirmation of the disappearance of bulging: the intra-abdominal pressure resumed to 15 mmHg with a flow rate of 6 L/m to confirm the disappearance of groin bulging (Fig. 2D). Then the contralateral processus vaginalis was examined and closed simultaneously by using PIRS, if present.

## Results

**Figure 4:**
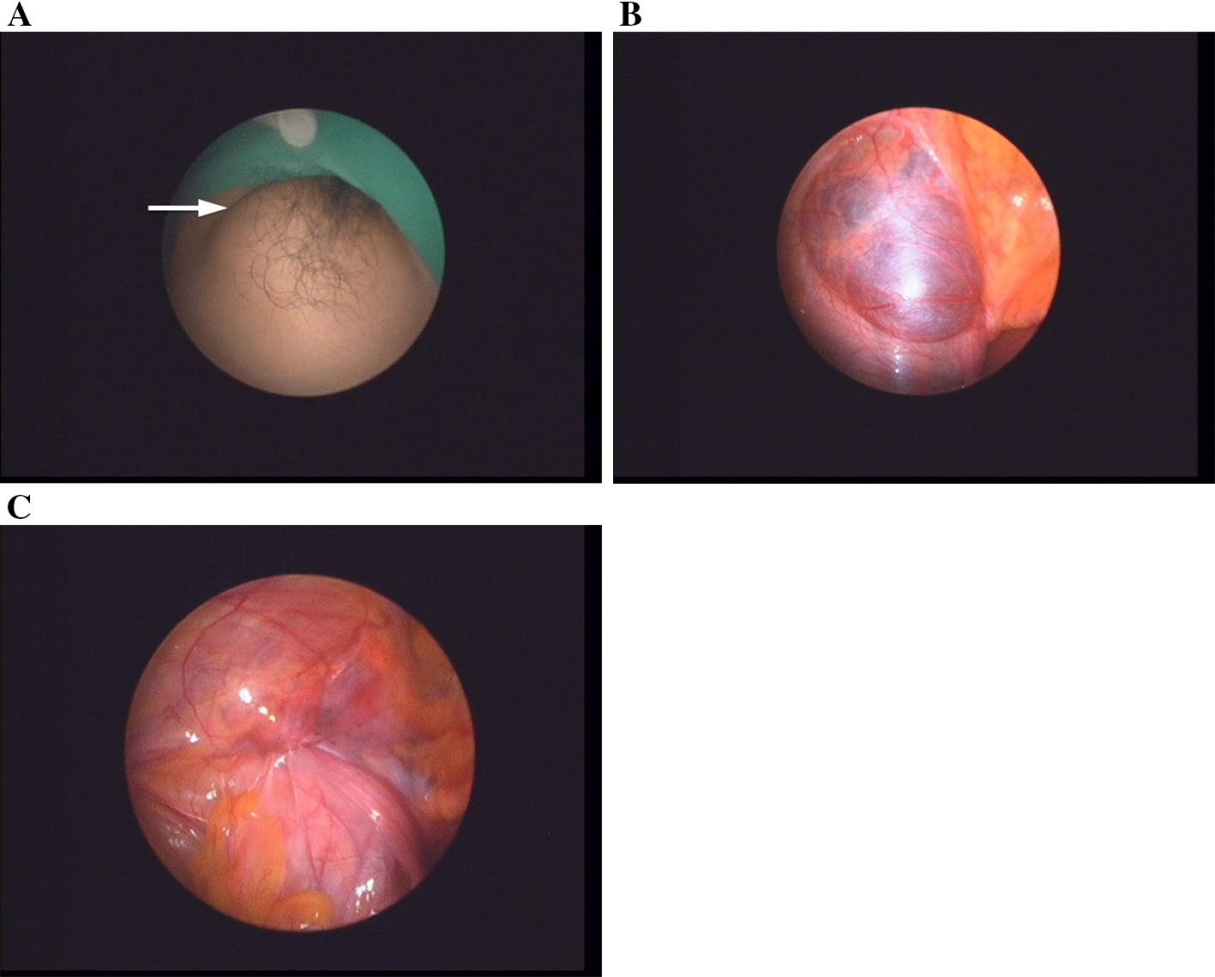
Not all groin bulges are inguinal hernias. (**A**) Left groin bulge was induced under pneumoperitoneum (white arrow). (**B**) An overt swelling mass noted alone the course of the inguinal canal. However, no internal ring opening and no posterior wall defect was found. (**C**) At the open surgery, an encysted hydrocele was noted and hydrocelectomy was performed. The groin bulge disappeared after surgery.

**Figure 5:**
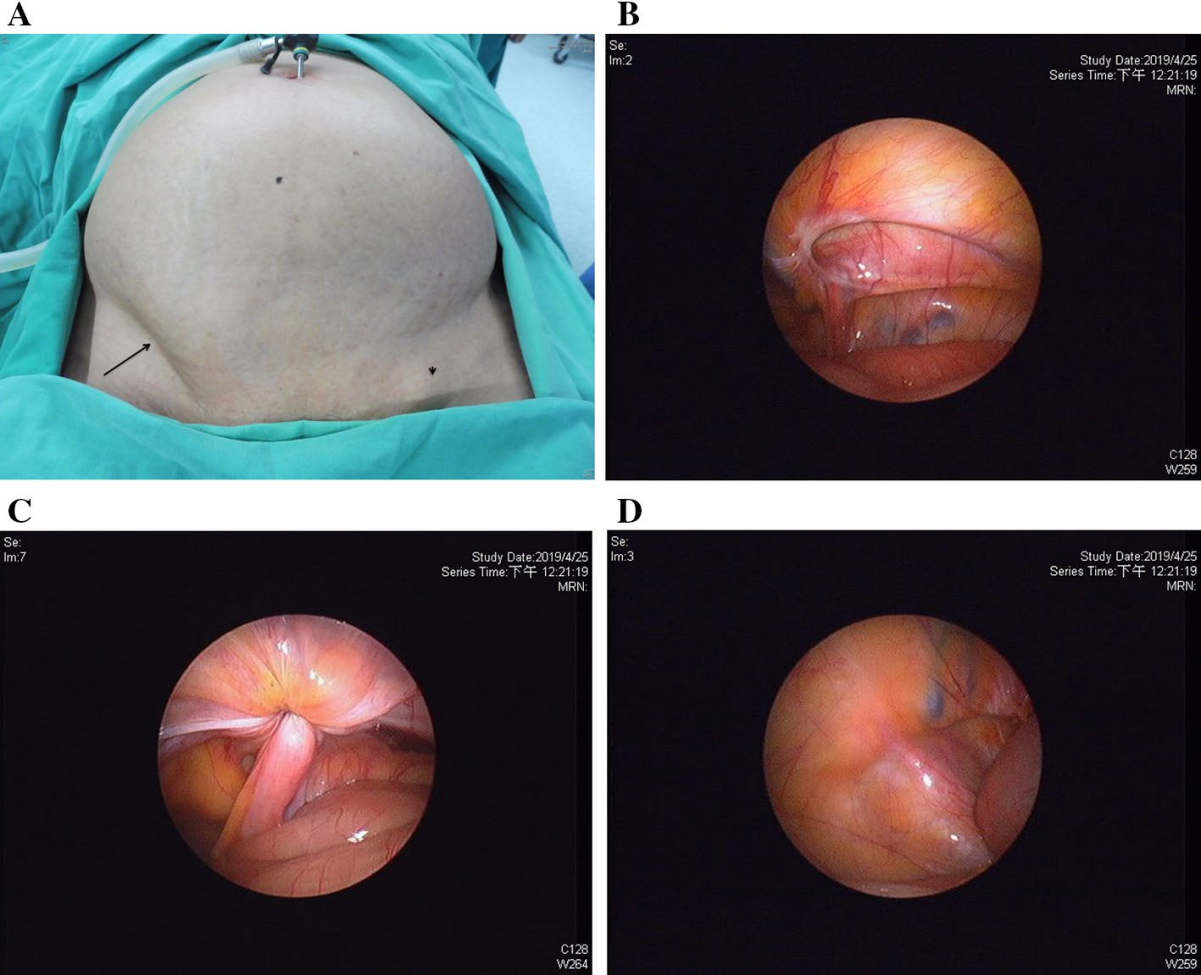
Incidental finding of an occult femoral hernia. (**A**) Under pneumoperitoneum, the right groin bulged alone the inguinal canal (arrow), while a small bulge also noted below the left inguinal ligament (arrow head). (**B**) A huge right internal ring opening, EHS L3, was noted. (**C**) The right internal ring opening was closed using PIRS. (**D**) The scopy also revealed a defect over the medial side of left femoral canal (arrow), which was compatible with the gross finding. This femoral hernia, EHS F1, was treated by open mesh repair.

Between April 2016 and Aug. 2019, there were 31 female adults who were diagnosed clinically as inguinal hernia and received single port laparoscopic examination (Fig. 1). One patient who was finally diagnosed as encysted hydrocele was excluded from the statistic studies (Fig. 4). For the 30 patients enrolled in this study, the median age was 38 years (range 20–88 years) with a majority (43%) of their onset periods between 1–6 months. The mean body weight was 54.0 ± 6.6 kg (range 43–73 kg) and the mean BMI was 21.4 ± 2.4 (range 17.5–27.8). All the 30 patients were IIHs with a total of 35 PIRSs performed. The number and percentage of patients with right: left: bilateral hernias were 17(56%): 11(37%): 2(7%), respectively. There were 3 CPPVs (10%) and 1 occult femoral hernia (3%) found during operation (Fig. 5). For the EHS classification of the 35 PIRSs, there are 14 L1 (40%), 17 L2 (49%), and 4 L3 (11%).

**Figure 6:**
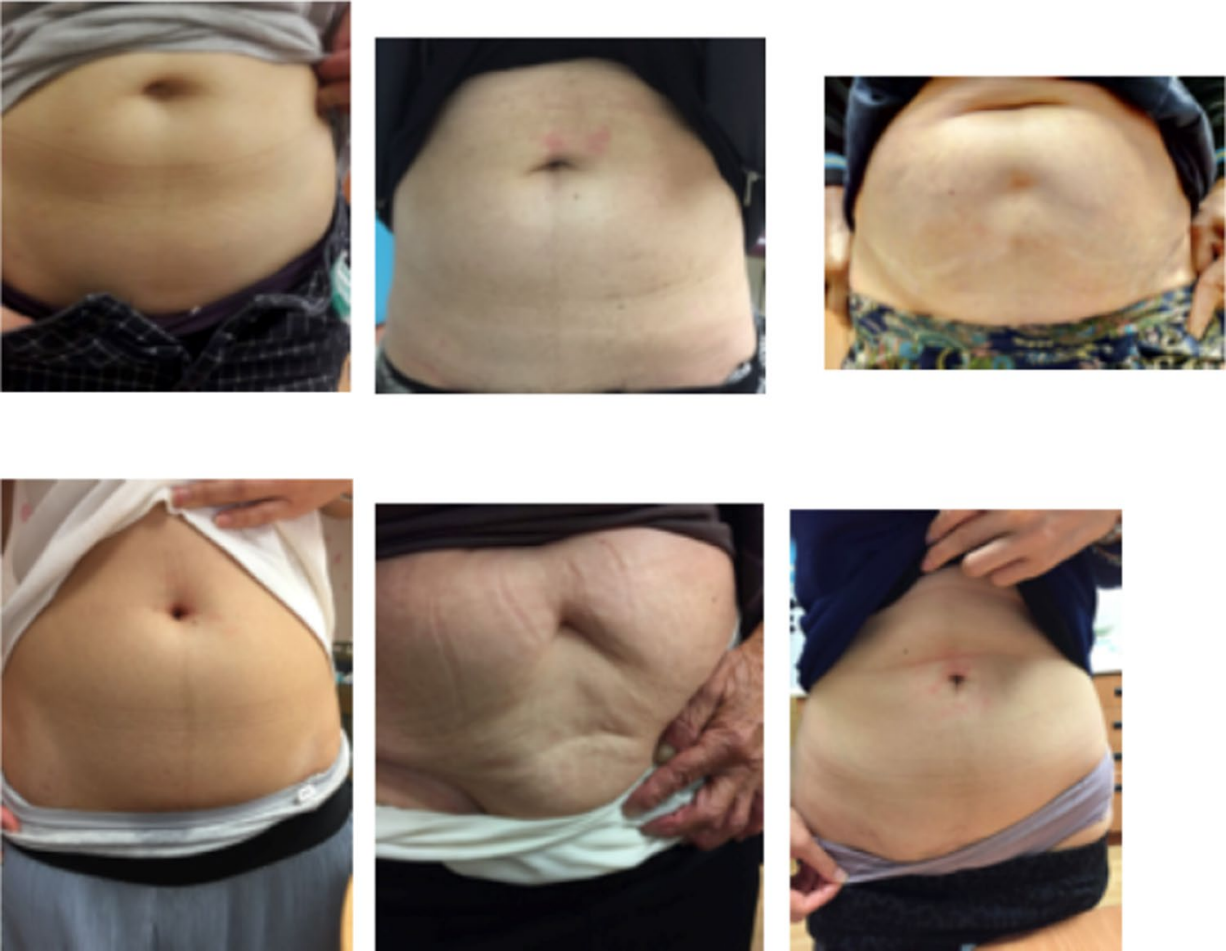
The post-operative cosmetic results were excellent. All the punctured sites were nearly invisible one week after PIRS.

The average operation time was 18 min for the unilateral and 30 min for bilateral hernias. All the hospital stays lasted less than 24 h. Most of the patients resumed their daily activities 2 days and the wounds were nearly invisible one week after operation (Fig. 6). There was no contralateral hernia developed after a mean follow-up of 13.6 months (range 3–29 months).

Two patients needed reoperation. One with an EHS L3 recurred 8 months postop due to stitch loosening. At the redo PIRS, two stitches were used to close the IRO. The other patient who suffered from postoperative groin pain for 4 months received open herniotomy and the previous stitch was removed to relieve pain. These two patients were doing well after reoperation and still in close follow-up.

## Discussions

Following the stepwise procedures under laparoscopy (Fig. 2), we can achieve at least three important aims of a hernia surgery. First, clarifying the classification of a hernia. Second, customizing the surgical technique according to classification. Third, ensuring the resolution of patient’s groin bulging after surgery. The procedures are started by the induction of groin bulging at a high intra-abdominal pressure and ended by ensuring the bulging resolved at the same pressure after surgery (Figs. 1, 2). In our series, one case was excluded from the statistic study due to non-hernia noted under laparoscopy (Fig. 4). All the other 30 cases were confirmed to be IIHs and received PIRS smoothly. We also found one occult femoral hernia and 3 CPPVs, which were treated by open mesh repair and contralateral PIRS, respectively. (Figs. 1, 5).

**Figure 7:**
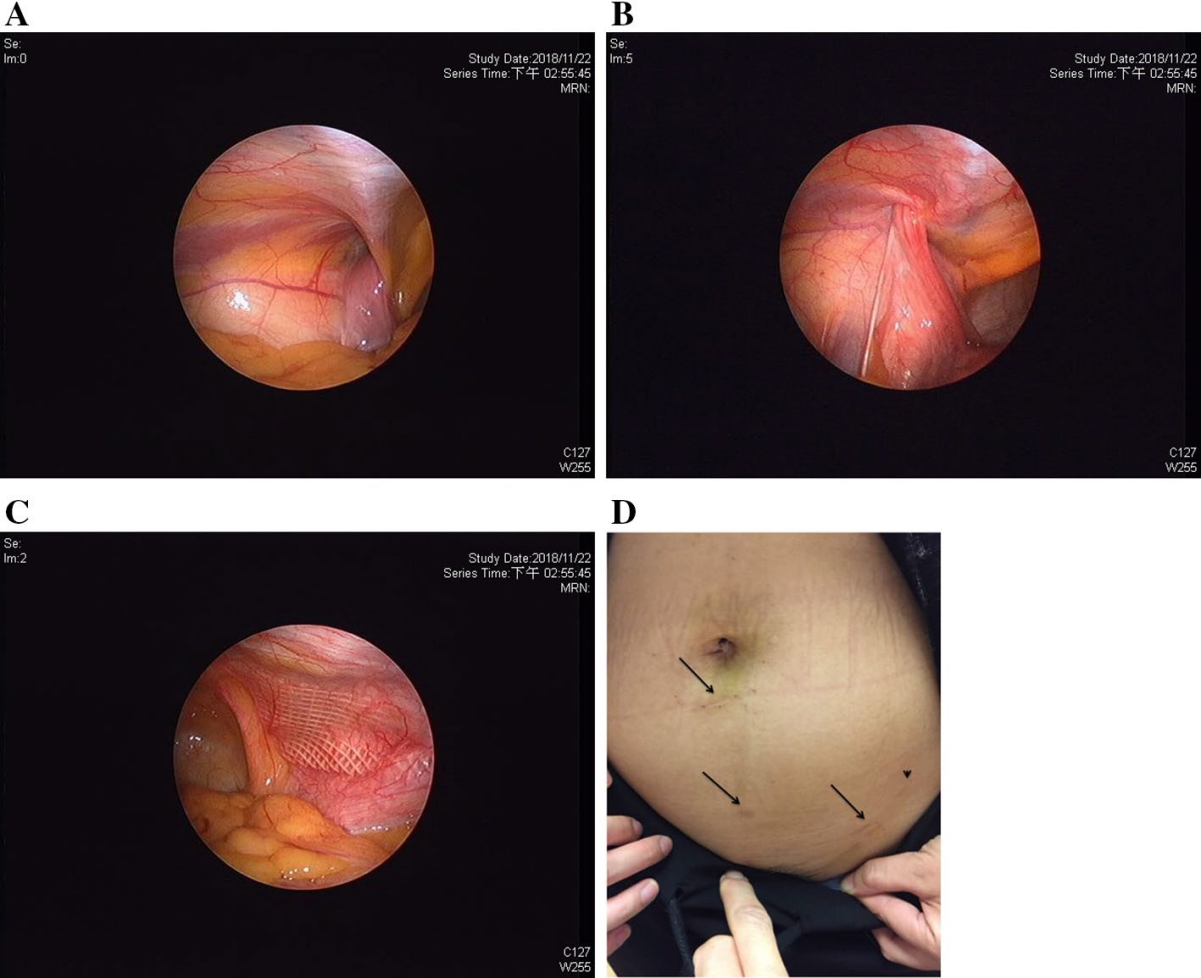
A comparison of post-operative pictures for the totally extraperitoneal hernia repair (TEP) and PIRS. This 39-year-old female had received right TEP and developed left heterochronic hernia 8 months later. (**A**) Laparoscopy showed an EHS L2 of the left internal ring opening. (**B**) Left PIRS was performed with minimal tissue trauma. (**C**) The previous TEP showed a huge mesh covering the wide area of right myopectineal orifice at the preperitoneal level. (**D**) Operative scars of the right TEP (arrow) and left PIRS (arrow head).

Although the preperitoneal mesh repair is recommended by the international guidelines for the repair of female IHs, a more aggressive tissue injury is necessary as the region of myopectineal orifice is widely dissected (Fig. 7). On the other hand, previous data showed that DIHs are very rare in women^1,14–16^. To customize the treatment of IHs according to their hernia types and minimize the tissue injury, we applied the technique of laparoscopic PIRS for the examination and repair of female IIH. Our data released here have covered a wide range of age groups (from 20 to 88 years) and onset periods (from < 1 month to > 2 years) of the female IHs. Interestingly, except for one encystic hydrocele, all the 30 female IHs were indirect type irrelevant of the patient’s age or onset period. Unlike the male adults who have a peak incidence of IHs in the seventies^17^, women seem to develop IHs earlier in their lives. In our series, up to 70% of patients were between the ages of 20–50 years. This is consistent with the clinical finding that most of the female IHs, like the pediatric groups, are indirect type.

Clarification of the hernia type is very important for both research and clinical practice^18^. Unfortunately, no classification system has been designed to evaluate the patient’s hernia type before surgery. That is the reason why an individualized surgical plan according to hernia type is very difficult to achieve, and the mesh-based techniques are predominant because they cover most types of IH with excellent results. Sonographic study has been used to determine the types of IHs before surgery. However, the accurate rates reported in literatures were ranged between 45–100%, depending on the size, type, identification of the anatomical landmarks, and the operator’s experience^19–24^. On the other hand, the laparoscopy is a good instrument to visualize the intra-abdominal wall and evaluate the hernia type definitely. Renzulli et al.^25^ ever suggested a diagnostic laparoscopy to obtain an accurate preoperative Nyhus classification. We use the EHS classification system in this study as it is simple and recommended by the HerniaSurge Group^10,18^.

To determine the detailed EHS classification of each patient, we further developed a calibration method to measure the diameter of IRO under the laparoscopic inspection. The needle we used is attached with a scale, allowing us to calibrate the diameter of IRO as the needle passing through the lower hemi-circumference of internal ring (Fig. 2B). The incidence of EHS L1, L2, and L3 for the total 35 PIRSs we performed was 40%, 49%, and 11%, respectively. To our knowledge, this is the first time to assess the EHS classification using the single port laparoscopy.

In the adult group, incidence of CPPV was estimated between 12–22% by using the laparoscopic exploration^26–28^. Due to the high risk of developing a heterochronic hernia, the HerniaSurge Group suggested a concomitant repair for the CPPV found during laparoscopy^18^. In our series, three CPPVs (10%) and one occult femoral hernia (3%) were noted during the operation, all were repaired at the same time.

Although our laparoscopic stepwise procedure provides a promising technique to resolve the patient’s groin bulging, we do have one recurrent case (3%). This 62-year-old patient with an EHS L3 recurred 8 month postoperatively due to stitch loosening. At the redo PIRS, the IRO was closed using two non-absorbable stitches. The patient is being well after reoperation. To prevent this complication, we now applied two non-absorbable stitches over the IRO for the EHS L3 hernias.

Chronic postoperative inguinal pain (CPIP) is another important issue in hernia surgery. The reported incidence of CPIP varied from 0.7 to > 75% may be due to the consensus definition of CPIP still not reached^29,30^. Awareness and recognition of those inguinal nerves during surgery is the gold standard to prevent this complication^18^. However, it is impossible to identify the inguinal nerves during the PIRS. Thus these inguinal nerves may be included in the encircled stitch, leading to nerve injury after tying the stitch. We have a 34-year-old woman who suffered from CPIP over the region of labia majora for 4 months after surgery. As a mechanical injury over the ilioinguinal nerve was highly suspected, an open herniotomy was arranged. During the surgery, the previous suture stitch was removed and the patient’s CPIP resolved completely. This implicated that the CPIP induced by the minimally invasive PIRS is a reversible condition after the relief of stitch.

Patients’ history of previous caesarean section or pelvic endometriosis had not become an obstacle to the creation of pneumoperitoneum when we used direct trocar insertion technique^31,32^. Because the 3-mm, sharp-ended trocar enabled us to make a quick access into abdominal cavity and then safely create pneumoperitoneum under direct vision provided by laparoscope. In our experience, only one patient with an umbilical scar due to previous laparoscopic colectomy needed open method (Hasson) to access abdominal cavity. No complications were noted in patients treated by either laparoscopic or open method.

This is the first report that single-port laparoscopic PIRS was logically applied in customizing the treatment of IHs for female adults. Although we have a satisfied early outcome, the scarcity of case volume is the limitation of this retrospective study. However, we have learned some precious experiences from this initial report including the avoidance and management of recurrence and CPIP, which have never been encountered with in the author’s experience on children’s IH (> 500 of paediatric PIRSs). It also raises some important issues. For example, what is the real incidence of IIH vs. DIH in the female group? What is the long-term recurrent rate after PIRS? Will the DIH develop following the PIRS? Is PIRS better than the conventional herniorrhaphy for a female IH? More cases accumulation and further prospective studies are needed to address these questions.

## Conclusion

This modified single-port laparoscopic PIRS is feasible for the management of indirect inguinal hernias in the female adults.

## Data Availability

The datasets generated and analyzed during the current study are available from the corresponding author on reasonable request.
